# *Pseudonocardia carboxydivorans* in human cerebrospinal fluid: a case report in a patient with traumatic brain injury

**DOI:** 10.1186/s12879-017-2538-y

**Published:** 2017-07-06

**Authors:** Amalia Navarro-Martínez, Noelia Corominas, Caridad Sainz de Baranda, Ángel Escudero-Jiménez, Jorge Galán- Ros, Juan Antonio Sáez- Nieto, Javier Solera

**Affiliations:** 1 Internal Medicine Department, University General Hospital, C/ Hermanos Falcó s/n, 02006 Albacete, Spain; 2 Microbiology Department, University General Hospital, C/Hermanos Falcó s/n, 02006 Albacete, Spain; 3 National Center of Microbiology (ISCIII, Madrid), Madrid, Spain

**Keywords:** *Pseudonocardia carboxydivorans*, Human cerebrospinal fluid (CSF), Traumatic brain injury (TBI), *Carboxydobacteria*

## Abstract

**Background:**

Members of the genus *Pseudonocardia* have been widely reported and recovered from several ecosystems, such as soil samples and plant samples. *Pseudonocardia* bacteria colonize the microbial communities on the integument of fungus gardening ant species. We present the first documented case of *Pseudonocardia carboxydivorans* isolated in human cerebrospinal fluid (CSF). To the best of our knowledge, this is the first report of an human infection by *P. carboxydivorans.*

**Case presentation:**

A patient, who suffered a traumatic brain injury a month before, was admitted to this hospital due to gait alteration and cognitive disturbances. Culture of cerebrospinal fluid showed ramified, not acid-fast, Gram positive bacilli. The bacterium was identified by molecular methods as *P. carboxydivorans*.

**Conclusion:**

This is the first documented case of isolating *P. carboxydivorans* in human CSF in a case of probable meningitis. Further research is needed in order to determine its pathogenic role in human infections.

## Background

*Pseudonocardia carboxydivorans* is a *Carboxydobacteria. Carboxydobacteria* are a group of aerobic bacteria that can grow aerobically on CO as the sole source of carbon and energy [1]. A bacterial strain, Y8^T^, capable of oxidizing carbon monoxide, was isolated from a soil sample collected from a roadside in Seoul, Korea. Strain Y8^T^ containes MK-9 as the major menaquinone, which is different from major menaquinone reported previously in the genus *Pseudonocardia*, MK-8 (H_4_). DNA-DNA relatedness between strain Y8^T^ and the type strains of *P. alni* and *P. antarctica* was respectively 10 and 63%. Based on phylogenetic, morphological and chemotaxonomic evidence, it is proposed that strain Y8^T^ be classified as the type strain of a novel species, *P. carboxydivorans* sp.nov. [2].

Members of the genus *Pseudonocardia* have been widely reported and recovered from several ecosystems, such as soil samples and plant samples [2, 3]. *Pseudonocardia* bacteria colonize the microbial communities on the integument of fungus-gardening ant species [4]. *Pseudonocardia spp.* is a known antifungal commensal microorganism [5] and a higer abundance of this taxon might rather reflect the presence of fungal organisms in the distal airways. The bronchoalveolar lavage fluid (BAL) microbiota of rheumatoid arthritis (RA) was significantly less diverse and abundant compared to healthy controls. The genus *Pseudonocardia* were the only taxa over represented in RA BAL and correlated with higher disease activity and erosions [6]. To the best of our knowledge, this is the first report of a probable human infection by *P.carboxydivorans.* That is the first documented case of isolating *P.carboxydivorans* in human CSF.

## Case presentation

**Fig. 1:**
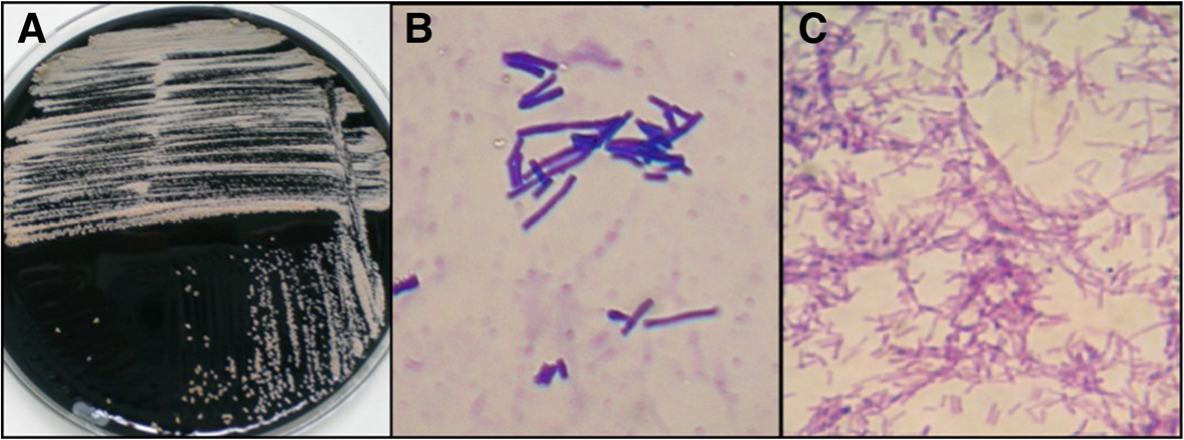
Microbiologic outcomes in cerebral spinal fluid. Panel **a** shows the culture of the specimen obtained from SCF the patient on selective medium for *Legionella* (BCYE) showing growth of white, dry and rough colonies after 7 days of incubation at 37 °C and 5% CO2 atmosphere. Panels **b** and **c** show the microscopic examination of the colonies by Gram and Kinyoun modified strain, showing the presence of ramified, not acid-fast, Gram-positive bacilli. The bacterium was identified by molecular methods of RNAr 16S sequencing, being finally identified as *Pseudonocardia carboxydivorans* in the National Center of Microbiology (ISCIII, Madrid, Spain)

A 67-year-old patient was admitted to this hospital due to gait alteration, cognitive disturbances and rapid clinical deterioration. The patient had a history of diabetes, asthma and a myocardial infarction 2 years earlier. Medications at the time of admission were acetylsalicylic acid, clopidogrel, glimepiride, pitavastatin and omeprazole. For the previous 2 years, he had intermittent syncope episodes. In one of them he suffered a fracture of the left tibia. Encephalogram, electrocardiogram (ECG), subcutaneos Holter, echocardiography and stress ECG were normal. A month before admission, hehad a new syncope episode while cultivating vegetables and he suffered a traumatic brain injury (TBI) with a left orbital and right occipital hematoma and he went to the Emergency Department of the Albacete General Hospital. A computed tomography (CT) showed a left subdural and retroorbital hematoma as well as a right occipital skull fracture. Neurologic examination was normal. A week after the injury, he suffered a respiratory infection with cough and yellow mucus and his primary care physician treated him with amoxicillin-clavulanic acid (875/125 mg every 8 h, 7 days, orally) with a consequent improvement of the respiratory symptoms. Then he was referred to the Internal Medicine Department for presenting continuous headache since the head injury and symptoms of progressive weakness in the lower limbs with instability while walking and confusion. The patient maintained normal vital signs and his body temperature was 36.5 °C. No skin lesions, lymph nodes or goiter were present. He carried a subcutaneous Holter device with no signs of local infection. Cardiopulmonary and abdominal explorations were normal. No edemas were present and the pulses were palpable. He showed disorientation to time and place, but not person. He had a paresis of the left seventh cranial nerve. He had a minimal decrease in strength in the lower limbs. Both the proprioceptive and the tactile sensitivity were diminished in the lower limbs, and showed no sensitivity to vibration. The tendinous reflexes were diminished in the examination. The finger-to-nose testing was impaired. The Romberg test was positive. The patient had gait disturbance with widened base and inability for tandem gait. He was admitted to the Internal Medicine Department. Laboratory tests were performed. Routine hematological testing showed a total hemoglobin 14.4 g/dl and a normal leucocyte and platelet count.Erytrocyte sedimentation raye was 51 mm/1 h (normal range values 1–20 mm/ 1 h). Prothrombin time and partial –thromboplastin time were normal. All blood chemistry values were normal except the values for glucose and C-reactive protein (CRP). The glucose concentration was 200 mg/dl (normal range values 74–109 mg/dl), the CRP level was 11.8 mg/l (normal range values 0–5 mg/l). Blood and urine cultures were negative. Chest x-ray was normal. Serology for *Treponema pallidum, Coxiella burnetii, BrucellaBorrelia burgdorferi, HIV, Cytomegalovirus, Epstein Barr virus, Simple herpes virus, Toxoplasma, Mycoplasma pneumoniae* was negative for acute infection with no seroconversion. A contrast magnetic resonance was carried out which revealed a right occipital fracture with a left frontobasal hematoma, a small left subdural occipital hematoma, a right occipital fracture with laminar subdural hematoma and a frontobasal contusion. A thoraco-abdomino-pelvic CT was carried out with no pathological findings. An electromyography (EMG) showed a sensory and motor mixed polyneuropathy with a severe demyelinization component. Moreover the patient showed a diabetic peripheral polyneuropathy. A lumbar puncture was carried out and CSF was obtained with a cell count of 105 cells/mcl with 100% mononuclear cells, glucose 100 mg/dl, 1.44 g/l proteins. As for the microbiological study, Gram staining of acid-fast bacilli, as well as aerobic,anaerobic,fungi,*Mycobacteria* and *Brucella* culture,were negative. Due to a positive tuberculin skin test (purified protein derivative, 5 TU) of 28 mm, a urine culture for *Mycobacteria* and a polymerase chain reaction (PCR) as well as a culture for *Mycobacterium tuberculosis* in CSF were requested and were negative. Faced with the possibility of a disimmune process and, after receiving the results of the electromyogram, it was decided on a treatment with 0.4 mg/kg/day intravenous immunoglobulin for 5 days with improvement of the clinical symptoms, recovery of the tendinous reflexes and the disappearance of the gait alteration. A second lumbar puncture was requested to check the characteristics of the fluid during treatment. The white blood cell count was 28 cells/mcl, predominantly mononuclear, no malignant cells, 0.88 g/l protein and adenosine deaminase (ADA) 5.9 u/l. The cytology of CSF showed lymphoplasmacytosis. In the microbiological analysis of CSF, Gram staining showed a few polymorphonuclear leukocytes and absence of microbial flora. The aerobic culture was performed on blood agar medium, chocolate agar medium and selective medium for *Legionella* (BCYE). After 7 days of incubation at 37 °C and 5% CO_2_, the culture showed growth of white, dry and rough colonies (Fig. 1, Panel a), which were presumably identified as *Actinomyces spp.* by microbiological routine testing such as the Gram stain (Panel B) and Kinyoun stain (Panel C), showing the presence of ramified, not acid-fast, Gram-positive rods..The bacterium was identified by molecular methods of rRNA 16S sequencing,as *Pseudonocardia carboxydivorans* in the National Center of Microbiology (ISCIII, Madrid, Spain). The isolate was identified by means of 16 s rRNA sequence analysis using a previously reported method [7] The 1434 bp fragment obtained from the isolate showed a similarity of 99.7% with *P. carboxydivorans* (GeneBank accession numbers FJ547123, NR044092 and others). When using E -test, the strain was sensitive (MICs, μg/ml) to all antimicrobials tested: ciprofloxacin (0.12), amikacin (0.5), trimethoprim-sulfamethoxazole (2), linezolid (0.25), imipenem (0.12), amoxycilin-clavulanic (1), cefotaxime (0.5) and erythromycin (2) Treatment with trimethoprim-sulfamethoxazole was initiated (20 mg/Kg every 6 h) intravenously. He did not receive any other antibiotic treatment added.Once this germ was isolated and to complete the study, a third lumbar puncture was carried out (Table 1). The CSF flow cytometry was normal. Besides, a biopsy of the bone marrow and a karyotype were conducted, which were normal, and a PET-CT showed the absence of metabolically significant localization at any level.

**Table 1:** Profile of laboratory results among the 4 CFS samples collected from initial^a^

Sample no	Date of sample collection (day /mo/yr)	Cell count cells/mcl	Cell type	ADA (u/l)	Proteins g/l	Glucose mg/dl	CSF culture result
1	24/05/2013	105	100% MN	ND	1.44	90	Neg (*Pseudonocardia* ND)
2	05/06/2013	28	100% MN	5.9	0.88	84	*P. carboxydivorans*
3	20/06/2013	10	100% MN	5.8	0.78	96	*P. carboxydivorans*
4	27/08/2013	1	1 red blood cell	ND	0.71	92	*P. carboxydivorans*

^a^ *ND* no data, *Neg* negative, *MN* mononuclear

The patient received 3-week regimen of trimethoprim-sulfamethoxazole 20 mg/Kg every 6 h intravenously followed by oral treatment with trimethoprim-sulfamethoxazole (800/160 mg twice a day). Two and 10 weeks after initiating treatment, new CSF control samples were taken, where *P. carboxydivorans* was isolated again; however, the characteristics of the liquid were normal. The duration of antimicrobial therapy was 10 months. The patient declined a new lumbar puncture in the absence of symptoms. Patient follow-up at 15 months has shown a favorable evolution, with no relapse of symptoms.

## Discussion

The genus *Pseudonocardia* was first established by Henssen in 1957 [1] and since then the genus description has been revised adding new species [2].

*Pseudonocardia* is a known antifungal commensal microorganism [4, 5] and a higher abundance of this taxon might rather reflect the presence of fungal organismsin the distal airways*.* A bibliographic search on Pubmed, Medline, Up to Date and other search platforms on pathologies caused by this germ rendered only microbiological description but no clinical cases in human. The genus *Pseudonocardia* were the only taxa over represented in RA BAL and correlated with higher disease activity and erosion [6] To the best of our knowledge, this is the first report of a probable human infection by *P.carboxydivorans.* That is the first documented case of isolating *P.carboxydivorans* in human CSF.

Patients with traumatic brain injury (TBI) are at a high risk for developing infections. Respiratory tract infections are the most common, with *Acinetobacter spp.* being the emerging pathogens. In a recent study, meningitis developed in 2% of the patients, whereas surgical site infections other than meningitis occurred in 4.25% [8]. Communication of the CSF with the environment was a major risk factor for the development of both SSI and meningitis.

The portal of entry of *P. carboxydivorans* in this patient remains speculative. *Carboxydobacteria* are a group of aerobic bacterias, which have been isolated in soil samples. The patient suffered a syncope episode while cultivating vegetables and he suffered a traumatic brain injury (TBI), but with no neurosurgical procedure or open fracture. *Pseudonocardia* could have developed in the soil he cultivated and get in contact with the patient in the traumatic brain injury. The respiratory tract infection might have been another gate of entrance, since *Pseudonocardia* is a well-known antifungal commensal and a higher abundance of this taxon might rather reflect the presence of fungal organism in the distal airways. But this patient not had any fungal infections, not breathing or rheumatic pathologies, so we though this way of entrance would be improbable.

Our patient suffered a TBI with a left orbital and right occipital hematoma that could had occasioned meningitis. He developed headache, confusion, gait abnormalities and focal neurological deficits. Even though a polineuropathy was previously suspected and was treated with intravenous inmunoglobulin, we believe that the isolation of the germ in the CSF had a pathogenic role. After isolating the germ in the CSF we considered the possibility that it may have been the cause of the clinical symptoms shown by our patient and that its entryway was the head injury prior to his admission. *P. carboxydivorans* was the only microorganism isolated in the CSF. The bacterium was identified by molecular methods of rRNA 16S sequencing in the National Center of Microbiology (ISCIII, Madrid, Spain). Te patient was treated with trimethoprim-sulfamethoxazole according to the antibiogram and also because of the high level of this antibiotic in the CSF. The evolution was good but we could not verify the sterility of the CSF because the patient did not agree to a new lumbar puncture due to the fact that he was feeling well. To our knowledge, this is the first documented case of isolation of *P. carboxydivorans* in human cerebrospinal fluid.

## Conclusion

This is the first documented case of isolating *P. carboxydivorans* in human CSF in a patient with a probable meningitis. Further research is needed in order to determine its pathogenic role in human infections.

## Abbreviations

BAL: Bronchoalveolar lavage fluid
CRP: C-reactive protein
CSF: Cerebrospinal fluid
CT: Computed tomography
ECG: electrocardiogram
EMG: Electromyography
MN: Mononuclear
ND: No data
Neg: Negative
RA: Rheumatoid arthritis
SSIs: surgical site infections
TBI: Traumatic brain injury

## References

[1] Henssen A (1957). Beiträge zur Morphologie und Systematik der thermophilen Actinomyceten. Arch Mikrobiol.

[2] Park SW, Park ST, Lee JE, Kim YM (2008). *Pseudonocardia carboxydivorans* sp. nov., a carbon monoxide-oxidizing actinomycete, and an emended description of the genus *Pseudonocardia*. Int J Syst Evol Microbiol.

[3] Li J, Zhao G-Z, Varma A, Qin S, Xiong Z, Huang H-Y, Zhu W-Y, Zhao L-X (2012). *Pseudonocardia xishanensis sp. nov*., an endophytic actinomycete isolated from the roots of Artemisia annua L. Int J Syst Evol Microbiol.

[4] Mueller UG, Ishak H, Sen R, Gutell RR (2010). Placement of attine ant-associated *Pseudonocardia* in a global *Pseudonocardia* phylogeny *(Pseudonocardiaceae,Actinomycetales):*a test of two symbiont-association models. Antonie Van Leeuwenhoek.

[5] Sen P (2009). Generalized antifungal activity and 454 screening of *Pseudonocardia* and *Amycolaptosis* bacteria in nests of fungus-growing ants. Proc Natl Acad SclU S A.

[6] Scher JU (2016). The lung microbiota in early rheumatoid arthritis and autoinmmunity. Microbiome.

[7] Drancourt M, Bollet C, Carlioz A, MartelinR GJP, Raoult D (2000). 16S ribosomal DNA sequence analysis of a large collection of environtmental and clinical unidentifiable bacterial isolates. J Clin Microbiol.

[8] Kourberti IS, Vakis AF, Papadakis JA, Karabetsos DA, Bertsias G, Filippou M, Iannou A, Neophytou C, Anastasaki M, Samonis G (2012). Infections in traumatic brain injury patients. Clin Microbiol Infect.

